# Long segment ureterectomy with tapered demucosalized ileum replacement of ureter for ureteral cancer: a case report and literature review

**DOI:** 10.3389/fonc.2024.1426003

**Published:** 2024-08-09

**Authors:** Zhifei Xie, Mingwen Liu, Shulian Chen, Wen Tang, Guobiao Liang, Jingyu Xu, Zeju Zhao

**Affiliations:** ^1^ Department of Urology, The Affiliated Hospital of Zunyi Medical University, Zunyi, Guizhou, China; ^2^ The Collaborative Innovation Center of Tissue Damage Repair and Regeneration Medicine of Zunyi Medical University, Zunyi, Guizhou, China

**Keywords:** ureteral carcinoma, kidney-sparing surgical, tapered demucosalized ileum, ileal-ureteral replacement, long segment ureterectomy

## Abstract

Radical nephroureterectomy (RNU) with bladder sleeve resection is currently the gold standard for the treatment of high-risk ureteral cancer. However, in certain special cases, such as bilateral upper tract urothelial carcinoma(UTUC), isolated and chronic kidney disease, and low-risk UTUC, kidney sparing surgery(KSS) may represent a viable alternative, though it remains highly challenging. The current KSS options for ureteral cancer include endoscopic treatment, segmental ureterectomy, total ureterectomy combined with kidney autotransplantation and nephrostomy. These methods are associated with significant disadvantages, such as a high risk of recurrence and vascular-related complications. On the basis of previous studies, we creatively proposed a surgical method of long segment ureterectomy with tapered demucosalized ileum(TDI) replacement of the ureter for ureteral cancer, and successfully performed this operation on a patient with ureteral cancer. The follow-up results showed that this surgical method provides good tumor control while preserving the patient’s renal function and improves the inherent defect of the ileal replacement of the ureter, which is a feasible choice for patients with ureteral cancer and kidney preservation.

## Background

Upper tract urothelial carcinoma (UTUC) includes renal pelvic carcinoma and ureteral carcinoma, accounting for 5%–10% of all urothelial carcinoma. Radical nephroureterectomy (RNU) with bladder sleeve excision has always been regarded as the gold standard for the treatment of high-risk UTUC (1). However, in special circumstances such as bilateral UTUC, isolated and chronic kidney disease, and low-risk UTUC, kidney sparing surgery (KSS) may be a reasonable alternative (2–5). Endoscopic treatment and segmental ureterectomy are KSS options for ureteral cancer, but there are incomplete nidus resection and a higher risk of tumor recurrence (6–9). Some case series studies have reported that total ureterectomy and kidney autotransplantation combined with nephrostomy can be used for the treatment of multifocal ureteral cancer (10–12). However, its application is limited by graft function and vascular complications (13). Long segment ureterectomy reduces the risk of recurrence of ureteral cancer by increasing the resection length of the diseased ureter (14). For ureteral defects resulting from long segment ureterectomy, ileal ureteral replacement has been designed to bridge larger ureteral defects and has been shown to be safe in some studies (15, 16). Nonetheless, ileal substitutes have some inherent drawbacks, such as large lumen, mucus secretion and metabolite absorption, which usually lead to related complications, such as metabolic acidosis, electrolyte disorders and calculi or mucus obstruction (17). To address these complications of ileal-ureteral replacement surgery, improved techniques have been proposed, including tapered bowel grafts, Yang-Monti technique, and ileal serotype myotubes, but only part of the issues are solved (18, 19). To comprehensively resolve these issues, we have innovated a technique that involves remodeling the ileum segment by reducing its diameter and stripping the mucosa to minimize mucosal secretion and absorption. Preliminary results from our preclinical animal studies have demonstrated the feasibility and safety of this approach, without the associated complications of traditional ileal-ureteral replacement (20). This study aims to report the outcomes of ureteral cancer cases treated using this novel surgical method, focusing on tumor recurrence, renal function, electrolyte balance, and the patency of the ileal-ureteral replacement.

## Case presentation

**Figure 1:**
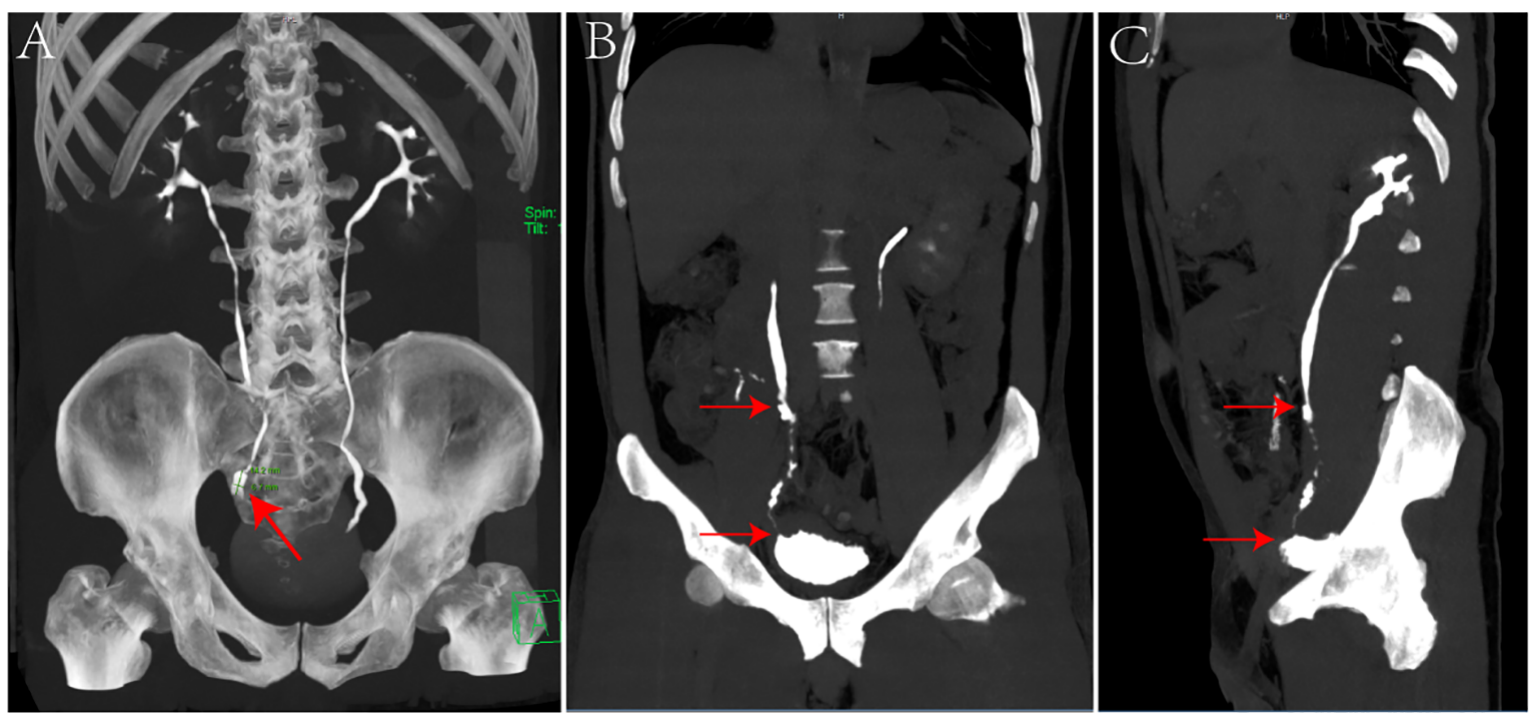
**(A)** Preoperative CTU scan revealing a 14.2x6.7mm swelling in the lower right ureter (red arrow, panel **A**). **(B, C)** CTU scans 40 months post-operation. **B** displays the anterior view and **C** the lateral view, indicating the anastomosis sites of the ileum segment with the ureter (upper arrow) and the bladder (lower arrow) (panels **B, C**).

**Figure 2:**
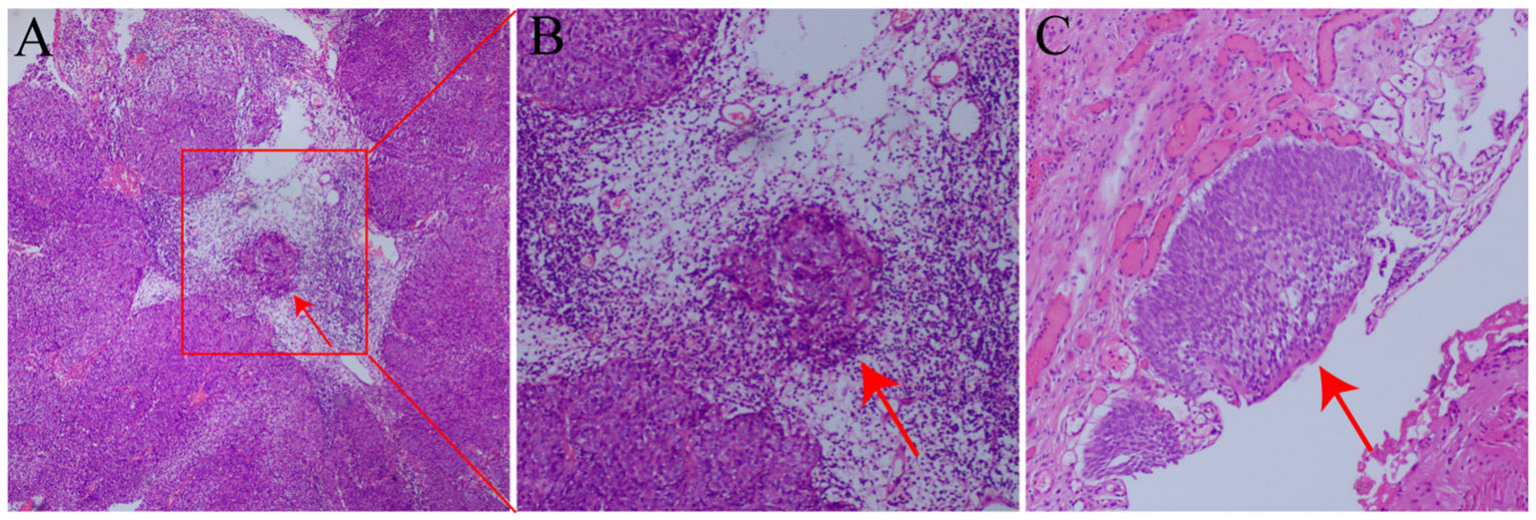
**(A)** HE stain of the preoperative biopsy showing high-grade non-invasive urothelial cancer. The red arrow denotes the cancer cell mass (magnification 40×, panel **A**). **(B)** Local magnified view of the cancer cell cluster from panel **A** (magnification 100×, panel **B**). **(C)** HE stain of the ureteral specimen post-surgical resection, indicating high-grade non-invasive urothelial carcinoma. The specimen’s cut end shows no tumor involvement, consistent with preoperative findings (magnification 100×, panel **C**).

**Figure 3:**
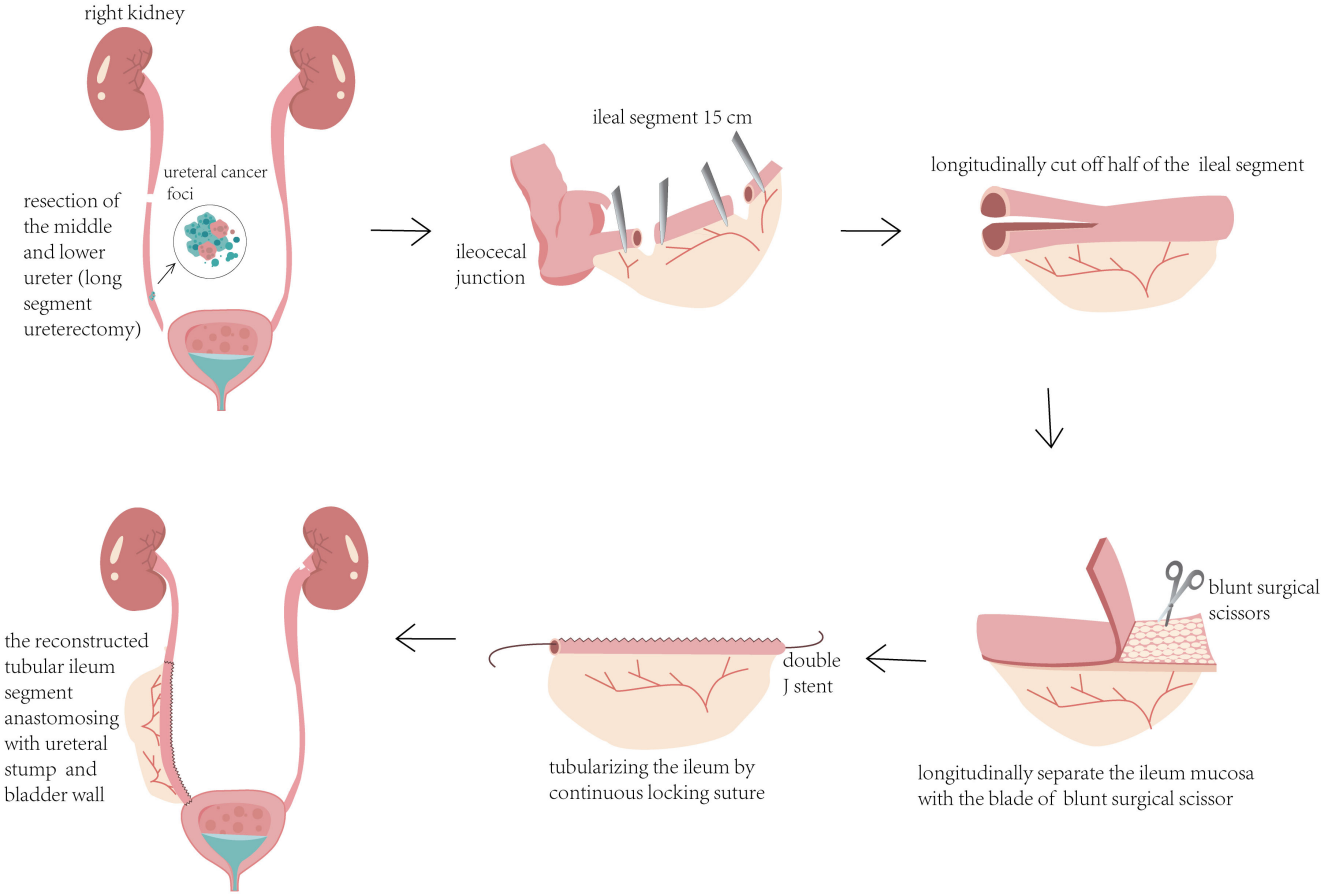
Schematic diagram of the surgical procedure.

**Figure 4:**
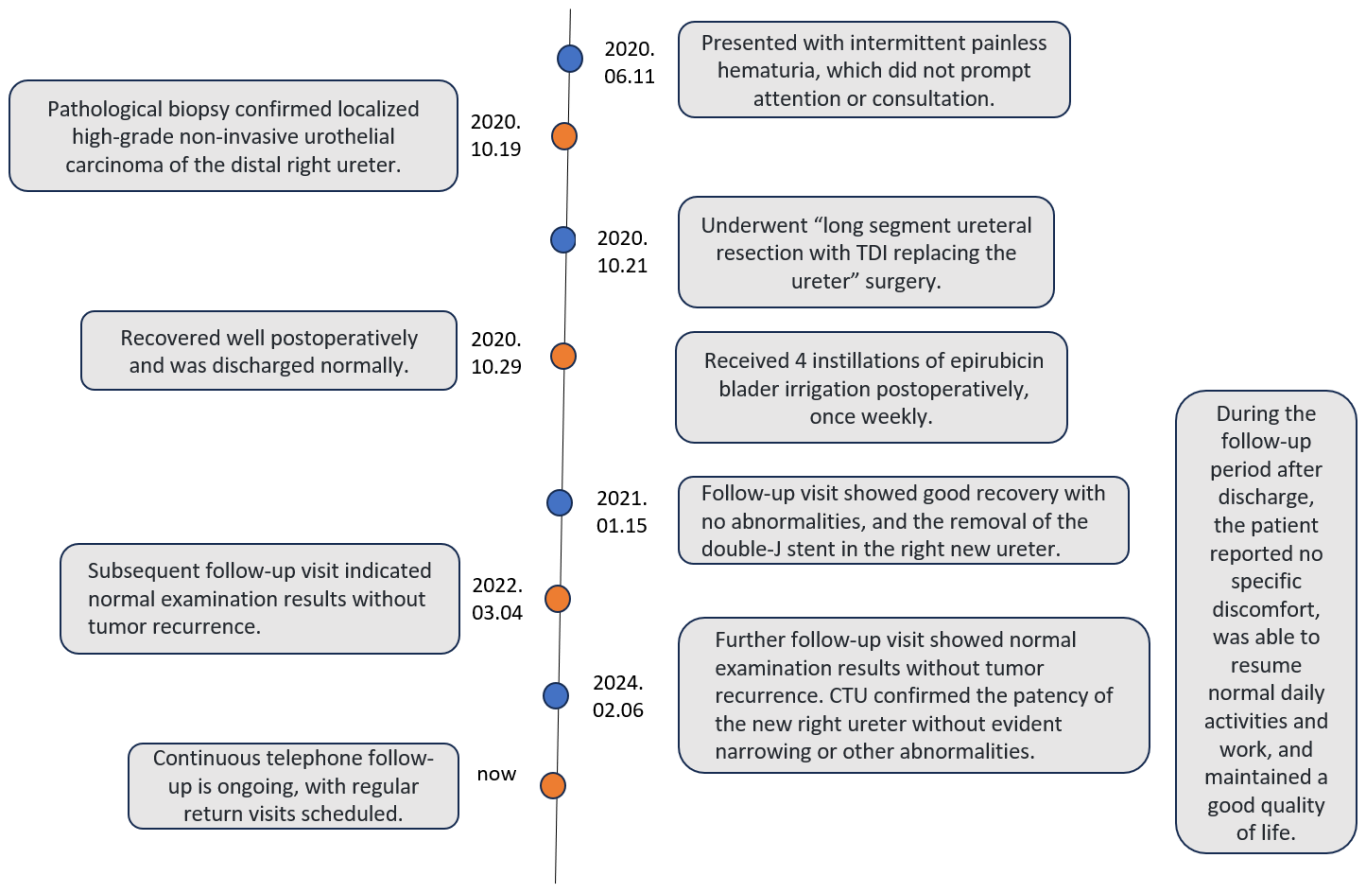
Disease diagnosis, treatment and follow-up time line.

The patient is a 42-year-old man employed as a construction worker, previously in good health with no history of other diseases or surgeries, and no family history of related cancers such as ureteral cancer. He had a history of heavy smoking (approximately 2 packs per day for 20 years) but no alcohol consumption. Four months prior to admission in October 2020, he began experiencing intermittent painless gross hematuria of unknown origin. Upon admission to our outpatient department, a general physical examination revealed no abnormalities, but routine urine analysis indicated a significant presence of red blood cells. To investigate the cause of hematuria, the patient underwent computed tomography urography (CTU), which revealed a swollen and dilated area measuring 14.2x6.7mm in the right lower ureter (**Figure 1A**). Subsequent ureteroscopy identified a prominent mass in the lower portion of the right ureter. A biopsy of the lesion confirmed a diagnosis of high-grade non-invasive papillary urothelial carcinoma (T1G1, **Figures 2A, B**). In October 2020, this patient underwent long segment ureterectomy with tapered demucosalized ileum (TDI) replacement for the ureter. The main surgical steps are described as follows(The schematic diagram of the surgical procedure is shown in **Figure 3**): make an incision beside the right rectus abdominis to open the abdominal cavity, enter the retroperitoneal cavity, expose the right middle and lower ureter and the vesicoureteral junction, perform the diseased ureter and bladder sleeve resection, the removed ureter is about 10cm long, cut off an ileum segment of about 15cm at least 15cm away from the ileocecum, longitudinally cut off half of the collected ileum, and longitudinally separate the ileum mucosa with the blade of blunt surgical scissors. After the mucosa separation is completed, A 12Fr rubber tube was placed in the cut ileum segment for support, and the ileum was closed into a tubular structure by continuous sewing with 3-0 absorbable thread. After that, double J stent (7Fr) was placed in the reconstructed ileum segment, and the proximal end was anastomosed with the ureteral stump and the distal end with the bladder wall along the peristalsis direction of the ileum segment. Two drainage tubes were placed around the anastomosis. The operation time was 380min, the intraoperative bleeding volume was 300ml, and the follow-up time was 40 months. Postoperative pathology confirmed local focal high-grade non-invasive urothelial carcinoma in the right ureter, with no tumor involvement at the resected ureteral end (**Figure 2C**). The patient’s recovery was uneventful; the catheter was removed one week after surgery, and he was discharged in good condition. Following discharge, he received four cycles of intravesical pirarubicin instillations once weekly. The double J stent was removed three months later. During the 40-month follow-up period, the patient remained in good health and resumed normal daily activities. Follow-up examinations indicated stable serum creatinine levels, glomerular filtration rate, and electrolyte balance (**Table 1**). CTU performed at 40 months post-surgery showed no tumor recurrence and no significant stenosis, mucus obstruction, calcification, or stone formation in the reconstructed ureter (**Figures 1B, C**). The diagnostic, therapeutic, and follow-up processes of this case are illustrated in **Figure 4**.

**Table 1:** Follow-up results of serum electrolyte, creatinine and GFR of patients.

Follow-up project	K+ (mmol/L)	Na+ (mmol/L)	CL- (mmol/L)	Scr (mg/dL)	GFR (ml/min)
Preoperative	4.0	138.0	110	0.94	75.86
1 week	4.1	137.5	107	0.89	70.25
16 months	4.2	140.0	108	1.04	65.77
40 months	3.9	138.2	108	0.96	68.04

## Discussion and conclusions

RNU with bladder sleeve resection for optimal tumor control in patients with UTUC is the current gold standard for the treatment of high-risk UTUC (1). However, removal of one side of the kidney results in excessive loss of renal function, increasing the risk of death and cardiovascular events (21). Moreover, advancing age raises the likelihood of diminished contralateral kidney function, impacting patient quality of life (22). In special cases such as bilateral UTUC, isolated and chronic kidney disease, and low-risk UTUC, KSS is a reasonable option (2–5). A middle-aged construction worker with normal bilateral renal function preoperatively presented with a solitary lesion in the right distal ureter. According to the European Association of Urology (EAU) guidelines, the patient’s UTUC was classified as low-risk, prompting consideration for renal preservation as the primary treatment approach. Given the patient’s occupational demands and strong preference for kidney preservation, KSS was deemed appropriate. However, achieving optimal oncologic control while preserving renal function in ureteral cancer remains challenging (23). We proposed an innovative surgical approach, long segment ureterectomy with TDI replacing the ureter, and animal experiments have been conducted to verify its safety and effectiveness (20).

Several studies have evaluated various surgical techniques for KSS in ureteral cancer. Endoscopic treatments and segmental ureterectomy are reserved for low-grade tumors with careful patient selection, offering comparable survival outcomes to RNU albeit with higher recurrence risks (5, 6). Total ureterectomy with kidney autotransplantation and nephrostomy achieves complete pathological ureter resection but is hindered by technical complexity and notable complications such as pseudoaneurysm, thrombosis, arterial stenosis, and hemorrhage (13).

Given the high recurrence risk associated with segmental ureterectomy, we employed extended ureteral resection to mitigate this risk (long segment ureterectomy). For substantial ureteral defects post-resection, the ileum serves as a suitable autologous tissue source widely applied in clinical practice (23). Nevertheless, conventional ileal-ureteral replacement surgery may lead to postoperative complications such as vesicoureteral reflux, metabolic acidosis, electrolyte imbalances, and calculi or mucus obstruction, attributable to mucosal secretion, waste absorption, and the large luminal diameter of the ileum (17). In order to overcome the problems caused by the large size of the ileal lumen, methods such as tapered intestinal grafts, Yang-Monti surgery have been proposed to reduce the area of the ileal lumen by cutting out the intestinal lumen and reconfiguring the bowel segments, which reduce mucus secretion but do not eliminate mucus secretion (18, 19). Attempts to mitigate the complications caused by the intrinsic absorption and secretion function of the ileal mucosa, it has been proposed to use the ileal serotype myotubes for ureteral replacement, and although the secretion and absorption of the ileum are greatly reduced, severe atrophy of the ileum occurs (24). In order to completely avoid the occurrence of complications after ileal-ureteral replacement, we modified the ileal-ureteral replacement on the basis of previous studies. For the first time, we reshaped the ileum segment by reducing the diameter of the ileum and stripping the mucosa. An animal experiment has been conducted to verify its safety and effectiveness (20). Notably, our technique involves partial excision of approximately half of the ileum’s luminal circumference away from the mesentery, followed by re-suturing to reduce luminal cross-sectional area. Mucosal stripping using blunt scissor blades was performed conservatively to minimize absorption and secretion while preserving muscularis mucosa integrity, avoiding potential ileal contractility issues associated with deeper mucosal removal. Some studies have shown that removing the intestinal muscularis mucosa and deeper mucosa will destroy the ileum nervous system and lead to its contraction (25).

We successfully performed the surgery, and the patients tolerated it well. Following the successful operation, we conducted regular follow-up examinations on the patients. Postoperative bladder irrigation with pirarubicin was performed to prevent metastasis and recurrence. During the follow-up, close attention was paid to the patients’ kidney function, internal environment, and the newly reconstructed ureter. In subsequent follow-up visits, there were no significant changes in the patients’ creatinine levels or electrolyte balance; however, there was a slight decrease in glomerular filtration rate compared to pre-surgery levels, likely due to the right ureteral surgery. Overall renal function remained good with no significant decline observed (**Table 1**). At the 40-month follow-up, the patient returned for a CTU review (**Figures 1B, C**). The anastomosis of the ileal segment with the ureter and bladder was successful. The reconstructed ureter on the right side was unobstructed, and both the ureter and bladder appeared normal without any filling defects. There were no signs of dilation in the renal pelvis or ureter, nor any abnormalities such as hydronephrosis, hydroureter, or contrast agent leakage, indicating good function of the reconstructed ureter in normal urine delivery to the bladder. Ureteroscopy revealed a smooth anastomosis of the ileal segment with no vegetations, strictures, or stone formations in the ureter or bladder. Throughout the follow-up period, the patient remained asymptomatic and was able to maintain a normal lifestyle and work routine with a good quality of life. The 40-month follow-up results confirmed no tumor recurrence or metastasis, normal renal function without significant decline, stable internal environment, and absence of traditional complications associated with ileal ureter replacement, such as electrolyte disorders, acid-base imbalances, or stone formation. These outcomes prove the effectiveness of the procedure in tumor control while preserving kidney function and avoiding complications linked with traditional ileal ureter replacement.

While this patient achieved effective tumor control, the efficacy and benefits of long segment ureterectomy for patients with ureteral carcinoma warrant confirmation through large prospective studies. The TDI procedure mitigates complications associated with traditional ileal ureter replacement; however, its technical demands, including longitudinal cutting of the ileal catheter and meticulous stitching to prevent urinary fistula formation, necessitate experienced surgeons.

For suitable patients with ureteral cancer, long segment ureterectomy with TDI is a feasible choice to replace ureteral surgery. Our experience suggests this approach maximizes tumor resection with favorable oncological outcomes while preserving diseased side kidney function and addressing inherent drawbacks of ileal-ureteral replacement. This study provides evidence supporting broader adoption of long segment ureterectomy with TDI in the management of ureteral cancer within the KSS framework.

## Data availability statement

The original contributions presented in the study are included in the article/supplementary material. Further inquiries can be directed to the corresponding authors.

## Ethics statement

The studies involving humans were approved by Ethics Committee of Affiliated Hospital of Zunyi Medical University. The studies were conducted in accordance with the local legislation and institutional requirements. The participants provided their written informed consent to participate in this study. Written informed consent was obtained from the individual(s) for the publication of any potentially identifiable images or data included in this article.

## Author contributions

ZX: Writing – original draft, Writing – review & editing. ML: Conceptualization, Data curation, Writing – review & editing. SC: Investigation, Methodology, Writing – review & editing. WT: Data curation, Writing – review & editing. GL: Formal analysis, Writing – review & editing. ZZ: Writing – review & editing, Investigation, Methodology, Supervision, Validation. JX: Writing – review & editing, study, Methodology, Supervision, Validation.

## References

[1] Rouprêt M Seisen T Birtle AJ Capoun O Compérat EM Dominguez-Escrig JL. European association of urology guidelines on upper urinary tract urothelial carcinoma: 2023 update. Eur Urol. (2023) 84:49–64. doi: 10.1016/j.eururo.2023.03.013 36967359

[2] Matsuda Y Inoue T Maeno A Koizumi A Yamamoto R Nara T. A patient with synchronous bilateral low-grade upper tract urothelial carcinoma who underwent nephroureterectomy and total ureterectomy with ileal ureteric replacement. Int Cancer Conf J. (2020) 9:82–7. doi: 10.1007/s13691-020-00402-w PMC7109248 32257759

[3] Seisen T Peyronnet B Dominguez-Escrig JL Bruins HM Yuan CY Babjuk M. Oncologic outcomes of kidney-sparing surgery versus radical nephroureterectomy for upper tract urothelial carcinoma: A systematic review by the EAU non-muscle invasive bladder cancer guidelines panel. Eur Urol. (2016) 70:1052–68. doi: 10.1016/j.eururo.2016.07.014 27477528

[4] Banerji JS George AJP. Total ureterectomy and ileal ureteric replacement for TCC ureter in a solitary kidney. Can Urol Assoc J J Assoc Urol Can. (2014) 8:E938–940. doi: 10.5489/cuaj.2255 PMC4277541 25553174

[5] Paciotti M Alkhatib KY Nguyen D-D Yim K Lipsitz SR Mossanen M. Is segmental ureterectomy associated with inferior survival for localized upper-tract urothelial carcinoma of the ureter compared to radical nephroureterectomy? Cancers. (2023) 15:1373. doi: 10.3390/cancers15051373 36900166 PMC10000204

[6] Suriano F Brancato T. Nephron-sparing management of upper tract urothelial carcinoma. Rev Urol. (2014) 16(4):21–8. PMC4004281 24791152

[7] Fang D Seisen T Yang K Liu P Fan X Singla N. A systematic review and meta-analysis of oncological and renal function outcomes obtained after segmental ureterectomy versus radical nephroureterectomy for upper tract urothelial carcinoma. Eur J Surg Oncol. (2016) 42:1625–35. doi: 10.1016/j.ejso.2016.08.008 PMC7312551 27612412

[8] Yakoubi R Colin P Seisen T Léon P Nison L Bozzini G. Radical nephroureterectomy versus endoscopic procedures for the treatment of localised upper tract urothelial carcinoma: a meta-analysis and a systematic review of current evidence from comparative studies. Eur J Surg Oncol. (2014) 40:1629–34. doi: 10.1016/j.ejso.2014.06.007 25108813

[9] Hendriks N Baard J Beerlage HP Schout BMA Doherty KSG Pelger RCM. Survival and long-term effects of kidney-sparing surgery versus radical nephroureterectomy on kidney function in patients with upper urinary tract urothelial carcinoma. Eur Urol Open Sci. (2022) 40:104–11. doi: 10.1016/j.euros.2022.04.007 PMC9142752 35638087

[10] Janssen MWW Linxweiler J Philipps I Bütow Z Siemer S Stöckle M. Kidney autotransplantation after nephrectomy and work bench surgery as an ultimate approach to nephron-sparing surgery. World J Surg Oncol. (2018) 16:35. doi: 10.1186/s12957-018-1338-1 29463251 PMC5819675

[11] Pettersson S Brynger H Henriksson C Johansson SL Nilson AE Ranch T. Treatment of urothelial tumors of the upper urinary tract by nephroureterectomy, renal autotransplantation, and pyelocystostomy. Cancer. (1984) 54:379–86. doi: 10.1002/1097-0142(19840801)54:3<379::aid-cncr2820540302>3.0.co;2-u 6375852

[12] Cheng SD Li WQ Mu L Ding GP Zhang B Shen C. Application of totally extraperitoneal renal autotransplantation with Boari flap-pelvis anastomosis in upper urinary tract urothelial carcinomas treatment. Beijing Da Xue Xue Bao Yi Xue Ban. (2019) 51(4):758–63. doi: 10.19723/j.issn.1671-167X.2019.04.029 PMC7433500 31420636

[13] Eisenberg ML Lee KL Zumrutbas AE Meng MV Freise CE Stoller ML. Long-term outcomes and late complications of laparoscopic nephrectomy with renal autotransplantation. J Urol. (2008) 179:240–3. doi: 10.1016/j.juro.2007.08.135 18001789

[14] Pedrosa JA Masterson TA Rice KR Kaimakliotis HZ Monn MF Bihrle R. Oncologic outcomes and prognostic impact of urothelial recurrences in patients undergoing segmental and total ureterectomy for upper tract urothelial carcinoma. Can Urol Assoc J. (2015) 9:E187–92. doi: 10.5489/cuaj.2408 PMC4455638 26085878

[15] Wolff B Chartier-Kastler E Mozer P Haertig A Bitker M-O Rouprêt M. Long-term functional outcomes after ileal ureter substitution: a single-center experience. Urology. (2011) 78:692–5. doi: 10.1016/j.urology.2011.04.054 21741686

[16] Verduyckt FJH Heesakkers JPFA Debruyne FMJ. Long-term results of ileum interposition for ureteral obstruction. Eur Urol. (2002) 42:181–7. doi: 10.1016/S0302-2838(02)00266-X 12160591

[17] Pamecha Y Shelke U Patil B Patwardhan S Kini S. Use of ileum for complex ureteric reconstruction: Assessment of long-term outcome, complications, and impact on renal function. Urol Ann. (2018) 10:369–74. doi: 10.4103/UA.UA_5_18 PMC6194786 30386088

[18] Patil N Javali T. Application of the “Yang-Monti principle” in children with iatrogenic ureteral injuries. J Pediatr Urol. (2021) 17:543.e1–7. doi: 10.1016/j.jpurol.2021.04.022 34034956

[19] Bao JS He Q Li Y Shi W Wu G Yue Z. Yang-monti principle in bridging long ureteral defects: cases report and A systemic review. Urol J. (2017) 14(4):4055–61. 28670676

[20] Gu H Chen S Wu Y Shen L Luo Y Li X. Improved long ureteral reconstruction with ileum by longitudinal clipping and mucosal stripping: an animal study. Urol J. (2020) 17:198–203. doi: 10.22037/uj.v0i0.5330 31912471

[21] Go AS Chertow GM Fan D McCulloch CE Hsu C. Chronic kidney disease and the risks of death, cardiovascular events, and hospitalization. N Engl J Med. (2004) 351:1296–305. doi: 10.1056/NEJMoa041031 15385656

[22] Meyer JP Delves GH Sullivan ME Keoghane SR. The effect of nephroureterectomy on glomerular filtration rate. BJU Int. (2006) 98(4):845–8. doi: 10.1111/j.1464-410X.2006.06373.x 16978282

[23] Ou Y-C Hu C-Y Cheng H-L Yang W-H. Long-term outcomes of total ureterectomy with ileal-ureteral substitution treatment for ureteral cancer: a single-center experience. BMC Urol. (2018) 18:73. doi: 10.1186/s12894-018-0389-5 30170590 PMC6119331

[24] Ibrahim ME Ezzat MM Ezzat WM. The use of seromuscular tapered ileal tube in ureteral replacement: an experimental model. Int Urol Nephrol. (2010) 42:697–701. doi: 10.1007/s11255-009-9672-4 20013053

[25] Urbán D Marei MM Hajnal D Varga G Érces D Poles M. Mucosectomy disrupting the enteric nervous system causes contraction and shrinkage of gastrointestinal flaps: potential implications for augmentation cystoplasty. J Pediatr Urol. (2020) 16:20–6. doi: 10.1016/j.jpurol.2019.08.019 31761695

